# Refeeding syndrome: multimodal monitoring and clinical manifestation of an internal severe neurotrauma

**DOI:** 10.1007/s10877-020-00513-y

**Published:** 2020-05-04

**Authors:** Nina Sundström, Camilla Brorsson, Marcus Karlsson, Urban Wiklund, Lars-Owe D. Koskinen

**Affiliations:** 1 Department of Radiation Sciences, Biomedical Engineering, Umeå University, Umeå, Sweden; 2 Department of Anaesthesia and Intensive Care, Institution of Surgery and Perioperative Sciences, Umeå University, Umeå, Sweden; 3 Division of Pharmacology and Clinical Neuroscience, Department of Neurosurgery, Umeå University, Umeå, Sweden

**Keywords:** Refeeding syndrome, Obesity surgery, Neurotrauma, Cerebral autoregulation, Compensatory reserve, Multimodal monitoring

## Abstract

Refeeding syndrome (RFS) is a rare, potentially life-threatening, condition seen in malnourished patients starting refeeding. RFS may provoke seizures and acute encephalopathy and can be considered an internal severe neurotrauma in need of specific treatment. The objective was to describe course of disease, treatment and, for the first time, multimodal monitoring output in a comatose patient suffering RFS. After gastric-banding and severe weight loss, the patient initiated self-starving and was transferred to our intensive care unit (ICU) following rapid refeeding. At arrival, seizures, decrease in consciousness (GCS 7) and suspected acute encephalitis was presented. Serum albumin was 8 g/l. Intracranial pressure (ICP), invasive blood pressure and electrocardiography (ECG) were monitored. Pressure reactivity (PR_x_) and compliance (RAP) were calculated. The patient developed congestive heart failure, anuria and general oedema despite maximal neuro- and general ICU treatment. Global cerebral oedema and hypoperfusion areas with established ischemia were seen. ECG revealed massive cardiac arrhythmia and disturbed autonomic regulation. PR_x_ indicated intact autoregulation (−0.06 ± 0.18, mean ± SD) and relatively normal compliance (RAP = 0.23 ± 0.13). After 15 days the clinical state was improved, and the patient returned to the primary hospital. RFS was associated with serious deviations in homeostasis, high ICP levels, ECG abnormalities, kidney and lung affections. It is of utmost importance to recognize this rare syndrome and to treat appropriately. Despite the severe clinical state, cerebral autoregulation and compensatory reserve were generally normal, questioning the applicability of indirect measurements such as PR_x_ and RAP during neuro-intensive care treatment of RFS patients with cerebral engagement.

## Introduction

The refeeding syndrome (RFS) was described after World War II, where prisoners were refed after liberation, and as a result developed peripheral edema and neuropathy [1]. RFS is a potentially fatal complex condition, where fluid and electrolyte shifts occur in severely malnourished patients when incautious nutrition is undertaken [2]. When oral, enteral or parenteral nutrition is introduced, glycemia leads to an increase in insulin production and a reduction in glucagon secretion. The increased insulin level stimulates the synthesis of protein, fat and glycogen which results in reduced serum levels of phosphate, potassium and magnesium since these are necessary components in the synthesis process [3]. Additionally, sodium and fluid balance disorders are common [3]. The incidence of RFS is unknown, and there are no clear definitions of this condition [4, 5].

The NICE guidelines [6] state the primary risk factors for developing RFS, e.g. a BMI < 16 kg/m^2^, fast and unintentional weight loss and a low nutritional intake for more than 10 days. However, another factor also found to be significantly associated with the risk of RFS is chronic weight loss following obesity surgery [6–8]. The incidence of RFS following gastric-banding and other weight reducing surgical procedures is unknown, and although it has been found that RFS might affect the physiological functions of e.g. the cardiac and neurological systems, and even lead to sudden death [5], the reports and descriptions in scientific literature is scares. Since encephalopathy is a known complication following RFS, though rarely described, this patient was monitored multimodally in our intensive care unit (ICU). The objective of this study was to describe course of disease, treatment and, for the first time, state and changes of the cardiovascular and cerebrovascular systems during ICU care of a severely ill patient suffering from RFS.

### Case description

**Fig. 1:**
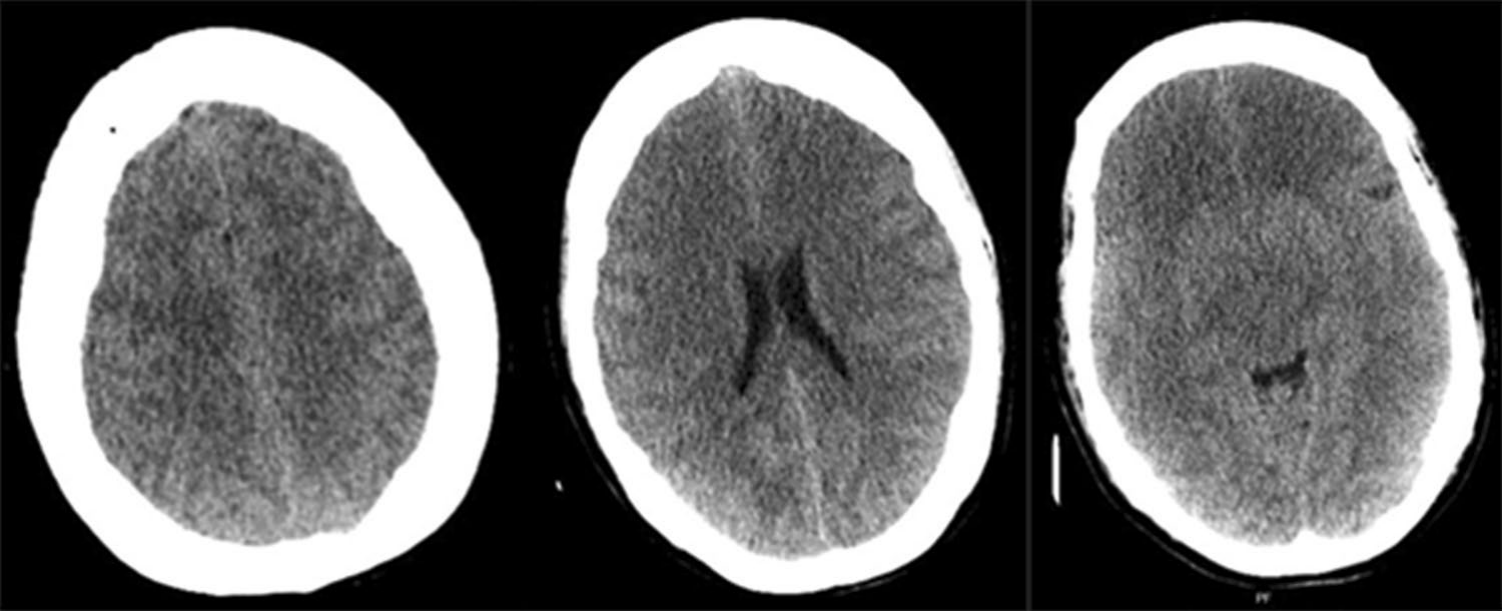
Initial CT scan showing general cerebral edema

A 49-year-old patient was admitted to a hospital due to abdominal pain. A gastric-by-pass operation had been performed eight years before this admission, and afterwards the patient developed an anorexic behavior and a severe malnutrition, resulting in the loss of 100 kg. Seven days prior to admission to the local hospital the patient terminated oral intake and was brought to the hospital by relatives. Body weight at admission was 145 kg, the patient was considered severely malnutritioned and was admitted to a surgical ward due to abdominal pain. Bilateral leg swelling was present, and s-albumin was 8 mg/L. Parenteral nutrition as well as enteral nutrition was started with 1800 kcal/day, and albumin was administrated. After three days the patient developed a stiff neck and decreased consciousness. Lumbar puncture was performed with no abnormal findings. Due to suspected septicemia antibiotics was started. The patient’s consciousness continued to decrease and intubation was necessary, hence the patient was referred to the local ICU. Caloric intake was reduced, and phosphate was administered. The next day the patient developed fasciculations in left hand, left part of the face, and anisocoria. A computed tomography (CT) scan was performed which revealed cerebral edema (Fig. 1). The patient was admitted to our university hospital.

At admission to the university hospital a cerebral intraparenchymal pressure device (Codman, DePuy Synthes, Raynham, MA, USA) instantly was inserted for intracranial pressure (ICP) monitoring and ICP immediately after insertion was reaching 60 mmHg (observed by the neurosurgeon LODK). Cerebral perfusion pressure (CPP) was calculated as the difference between mean MAP and ICP. At arrival in the ICU, initial arterial blood gas (ABG) revealed severe acidosis with pH 7.09, base excess − 19.3 mEq/L, standard bicarbonate 9.9 mmol/L and lactate 8.4 mmol/L. The patient was circulatory unstable with need of high doses of norepinephrine, and the oxygen demand was 70%. A cardiac echo examination revealed a dilated right ventricle and severe tricuspid leakage. To optimize contractility, and reduce afterload due to right ventricle failure, intravenous levosimendan and inhalation of sildenafil was initiated. Abdominal echo examination showed ascites but no portal thrombosis. Due to severe acidosis and anuria, hemodialysis was started. Shortly after this the patient developed atrial fibrillation, which was electrically cardioverted to sinus rhythm with frequent bursts of ventricular extrasystolic beats. Laboratory findings are presented in Table 1. Blood chemistry was analysed in the ISO 15189 accredited laboratory at Umeå University hospital. Plasma levels of albumin, phosphate, magnesium, vitamin B12 and folate were analysed by Roche ALBP, PHOS2, MG2, Elecsys Vitamin B12, Elecsys Folate III reagents on Cobas c8000 analysers. Zinc was analysed by Sentinel reagent on Cobas c8000 and INR by MediRox Owren's PT GHI 131 reagent on ACL TOP 700 analysers. Blood cell counts were analysed on Sysmex XE-5000 and blood gas analyses were performed using ABL 800 (Triolab, Gothenburg, Sweden).

**Table 1:** Patient laboratory characteristics

	Normal range	Day 1	Day 2	Day 3	Day 4	Day 5	Day 6	Day 7
Albumin (g/L)	36–45	27	22	23	19	21	20	27
Hemoglobin (g/L)	117–153	99	97	105	107	107	103	111
WBC (10E^9^ /L)	3.5–8.8	16.9	15.3	16.3	14.0	12.8	14.7	18.5
B12 (pmol/L)	141–489	> 1475						
Folate (nmol/L)	10–42	18						
Zinc (µmol/L)	9–15	6	6				9	
PT/INR	< 1.2	1.4	1.8	1.4	1.3	1.3	1.4	1.3
Phosphate (mmol/L)	0.8–1.5	1.8	0.85	0.54	0.76	1.02	m.v	1.19
pH	7.35–7.45	7.09	7.37	7.45	7.47	7.47	7.47	7.48
Base Excess (mmol/L)	-3 to 3	-19.3	-1.8	0.1	1.9	1.9	3.1	3.8
Standard bicarbonate (mmol/L)	21.0–26.0	9.9	22.8	24.8	26.4	26.4	27.3	28.0
Ionized calcium (mmol/L)	1.15–1.29	1.19	1.13	1.17	1.14	1.18	1.15	1.12
Sodium (mmol/L)	135–145	144	140	139	137	139	140	141
Potassium (mmol/L)	3.6–4.6	4.9	3.9	4.1	4.3	3.7	3.7	4.0
Magnesium (mmol/L)	0.7–0.95	1.26	0.94	0.86	0.80	0.79	m.v	0.71
Lactate ( mmol/L )	< 3.4	8.4	1.6	1.9	1.4	1.4	1.0	1.0

Laboratory values after admission to the University Hospital

Each value is the first value received on each day

* WBC* white blood count, *m.v*  missing value

Due to intractable ICP a ventriculostomy was performed. ICP decreased rapidly to 16 mmHg after cerebrospinal fluid diversion and intensifying the sedation by midazolam. Thiopental could not be administered due to cardiovascular instability. However, ICP increased rapidly again to 33 mmHg in the first three hours with a CPP between 43 and 55 mmHg. Within 24 h circulation and respiration were stabilized, but ICP was persistently increased with a CPP over 50 mmHg, and treatment with thiopental was initiated. A CT was performed revealing an intraventricular bleeding. After 96 h ICP finally decreased, thiopental was successively reduced and terminated on the 5th day after arrival.

High frequency data collection of ICP (125 samples/second), invasive mean arterial blood pressure (MAP, 125 samples/second) and electrocardiography (ECG, 500 samples/second) from the ICU system (Philips IntelliVue MX800, United States) was started in the afternoon on day 2, 35 h after admission (ixTrend, ixellence GmbH, Germany). Collection of ICP was continued until 03.20 on day 10, while collection of MAP and ECG were terminated 11 h later. CPP was calculated as the difference between mean MAP and ICP.

**Fig. 2:**
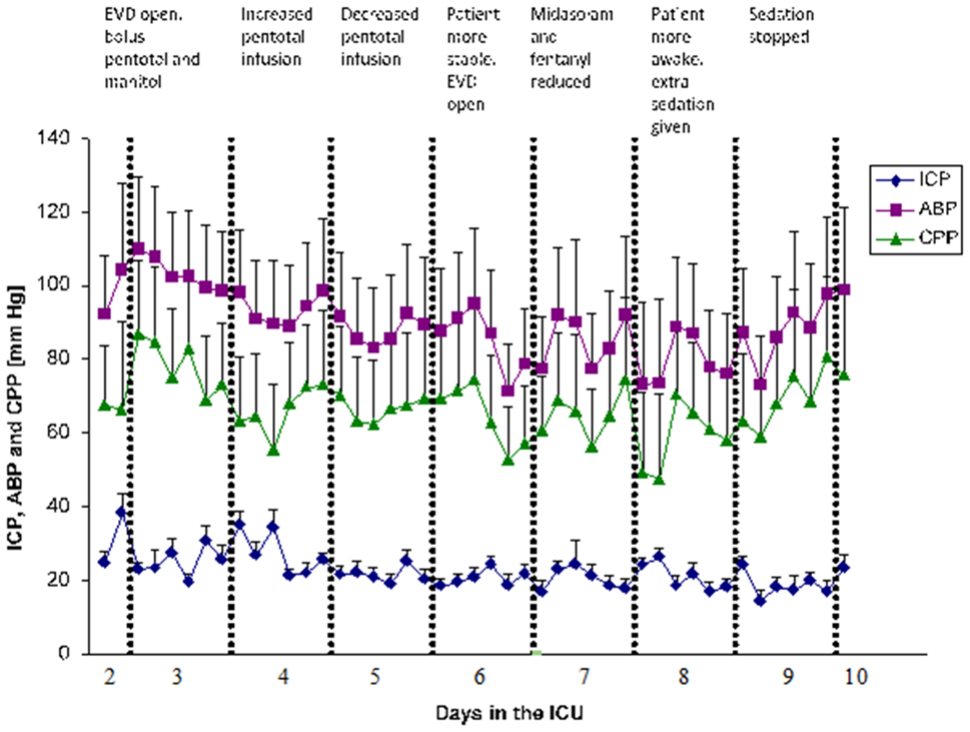
ICP, MAP and CPP (mean + SD) every four hours during day 2 to 10 in the ICU. Text above each day indicate main treatment strategy. *EVD* External ventricular drain

**Fig. 3:**
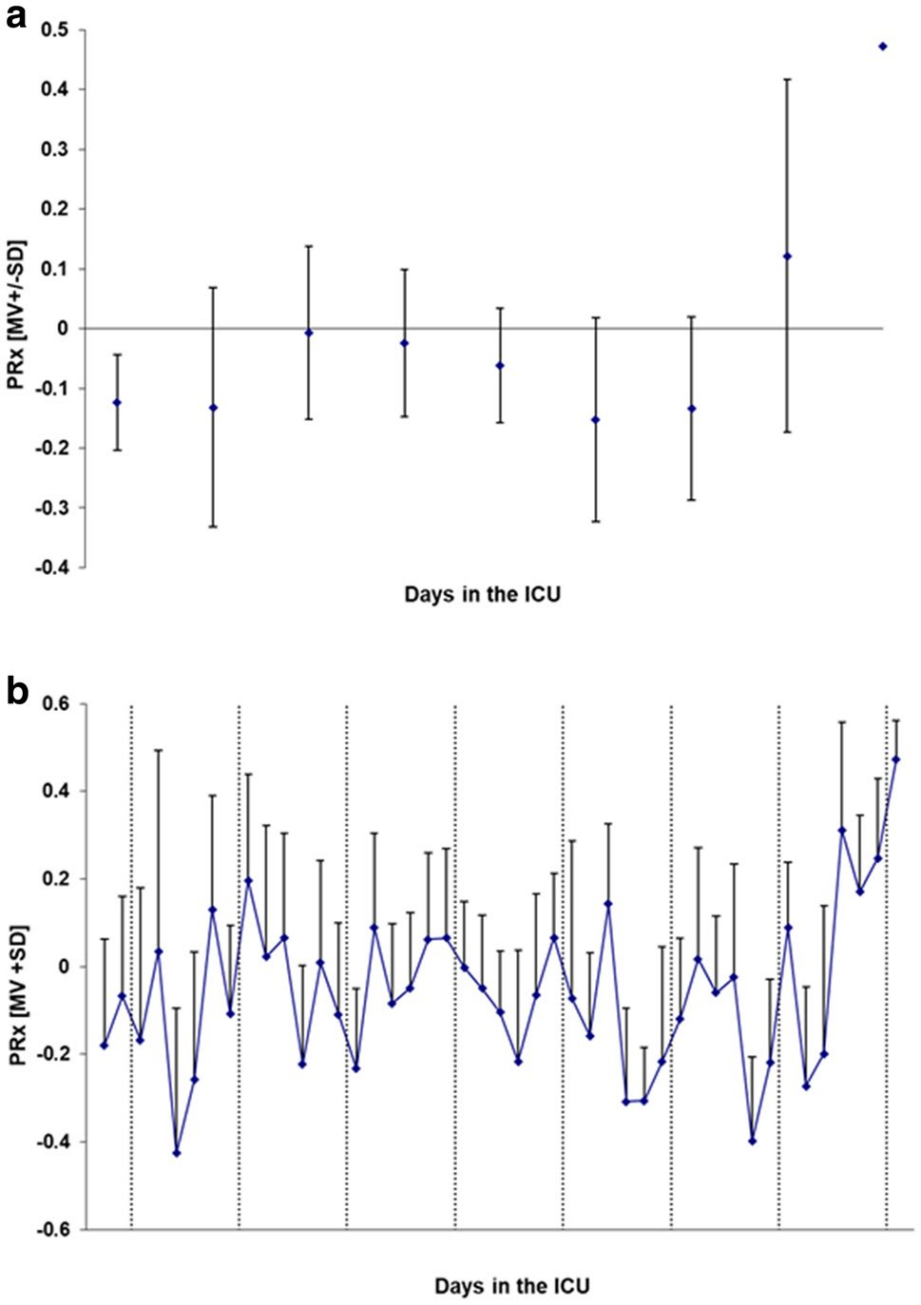
PRx (mean + SD) during day 2 to day 10 in the ICU **a** every 24 h, except for the first day where the mean is over the last 8 h, **b** every 4 h

**Fig. 4:**
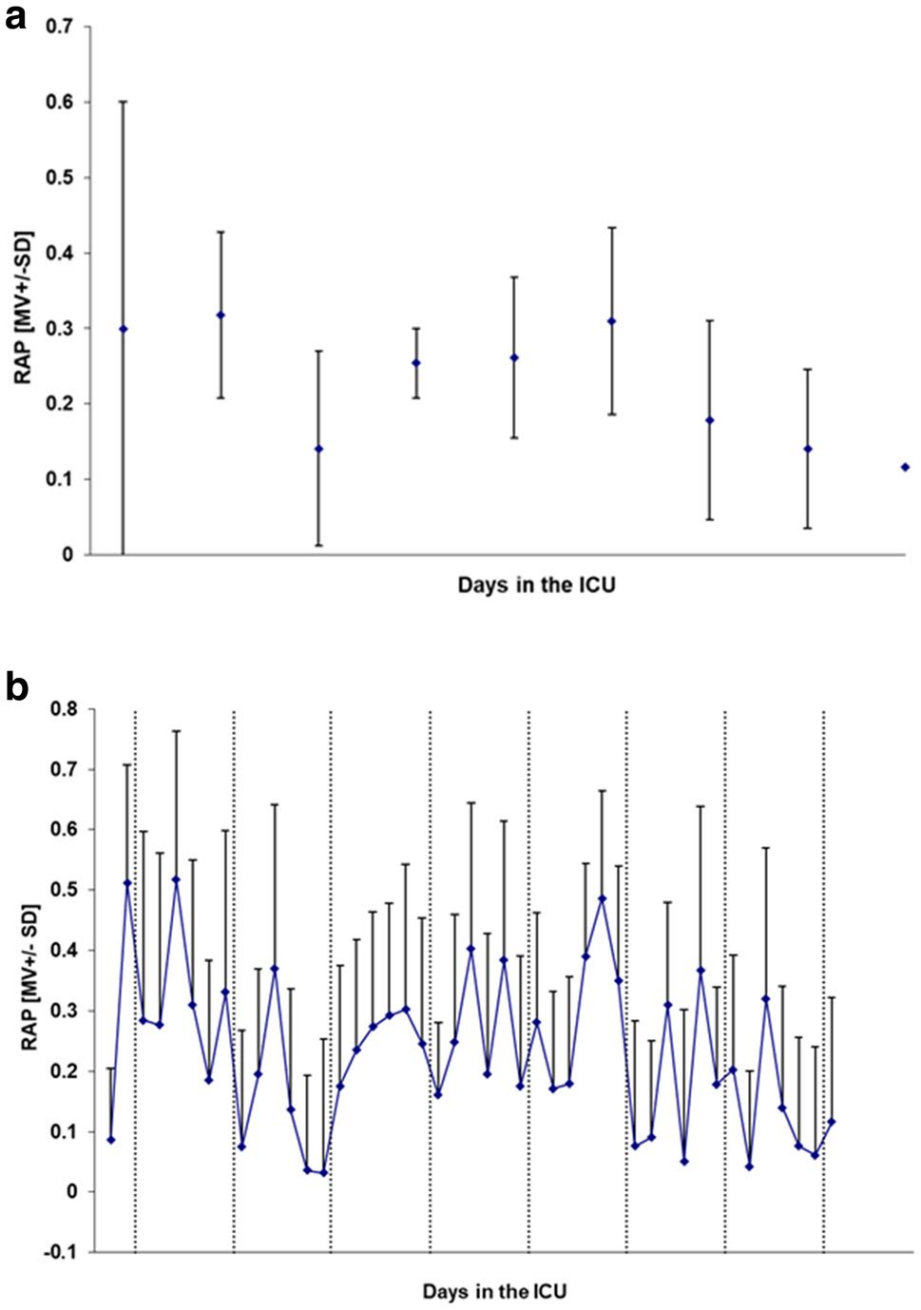
RAP (mean + SD) during day 2 to day 10 in the ICU **a** every 24 h, except for the first day where the mean is over the last 12 h, **b** every 4 h

From the multimodal high frequency data collection, periods of one-hour monitoring time were selected and analyzed every four hours. This was to exclude periods of treatment interventions and data collection disruptions due to e.g. x-ray examinations, and also to present the large amount of data in a condense way. ICP, MAP and CPP from day 2 to day 10 are shown in Fig. 2. The pressure reactivity index (PR_x_), considered as a measure of brain autoregulation, was calculated as the correlation coefficient between 40 consecutive ICP and MAP mean values over 6 s, i.e. a new PR_x_ index was calculated every four minutes (Fig. 3) [9]. Mean PR_x_ over the entire monitoring time was − 0.06 ± 0.18 (mean ± standard deviation (SD)). The compliance related index “RAP” (relationship between pulse wave amplitude and mean of ICP) was calculated on the same time basis, but as the correlation between the pulse wave amplitude of ICP and mean ICP over each time interval (Fig. 4) [10]. Mean RAP over the entire monitoring time was 0.23 ± 0.13.

Cardiac autonomic activity was assessed by power spectrum analysis of the heart rate variability (HRV). The recorded ECG of this patient showed frequent extra-systolic beats. However, arrhythmia-free 2-minute intervals were relatively common, whereas there were only a few 5-minute periods without excessive heart beats. Therefore, the 2-minute time interval was chosen as the time basis for the frequency domain analysis of HRV.

**Fig. 5:**
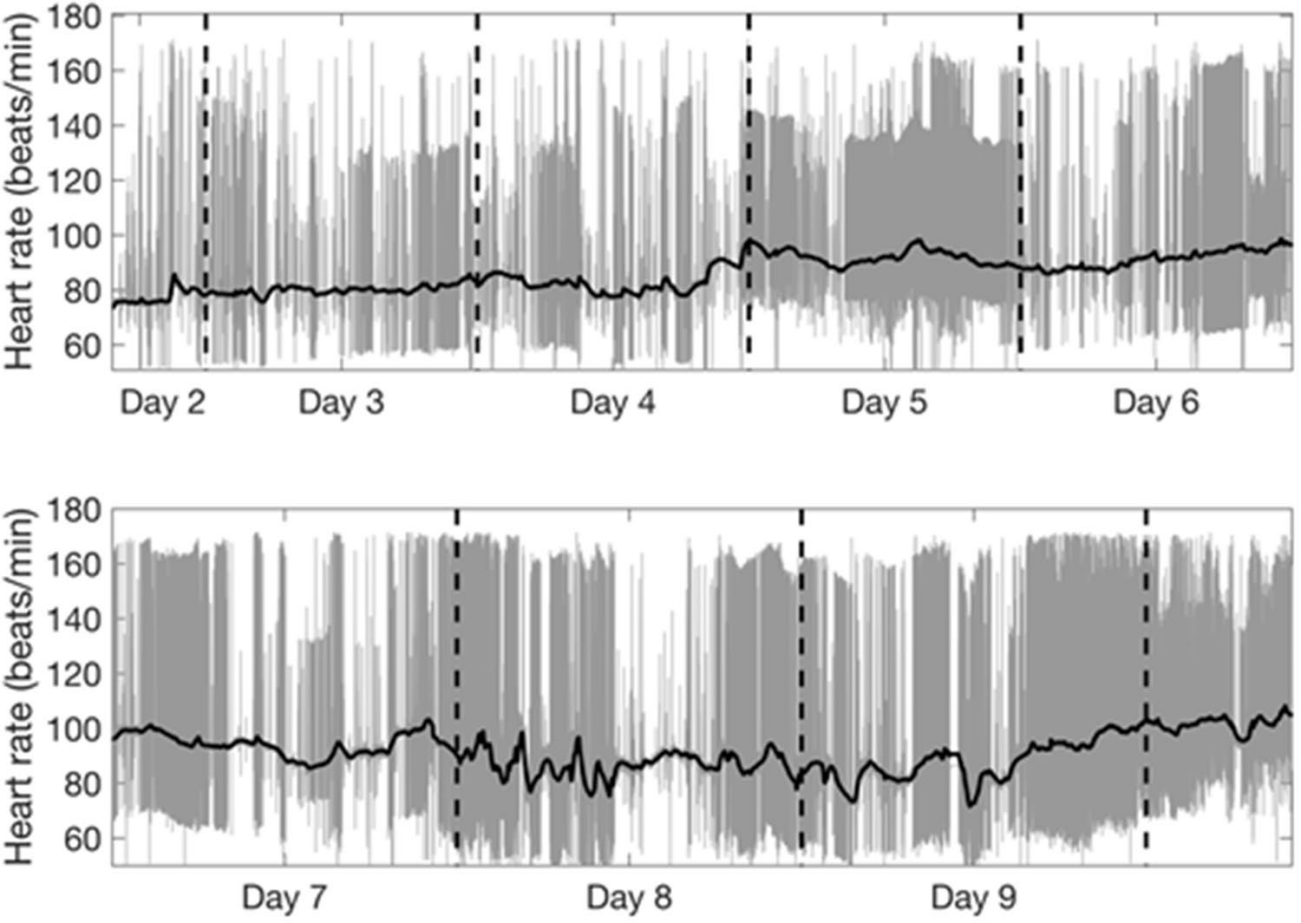
Heart rate during the multimodal recording in the ICU. The black line shows mean heart rate, whereas gray line shows the beat-to-beat changes illustrating how the frequency of arrhythmic heart beats varied over time

**Fig. 6:**
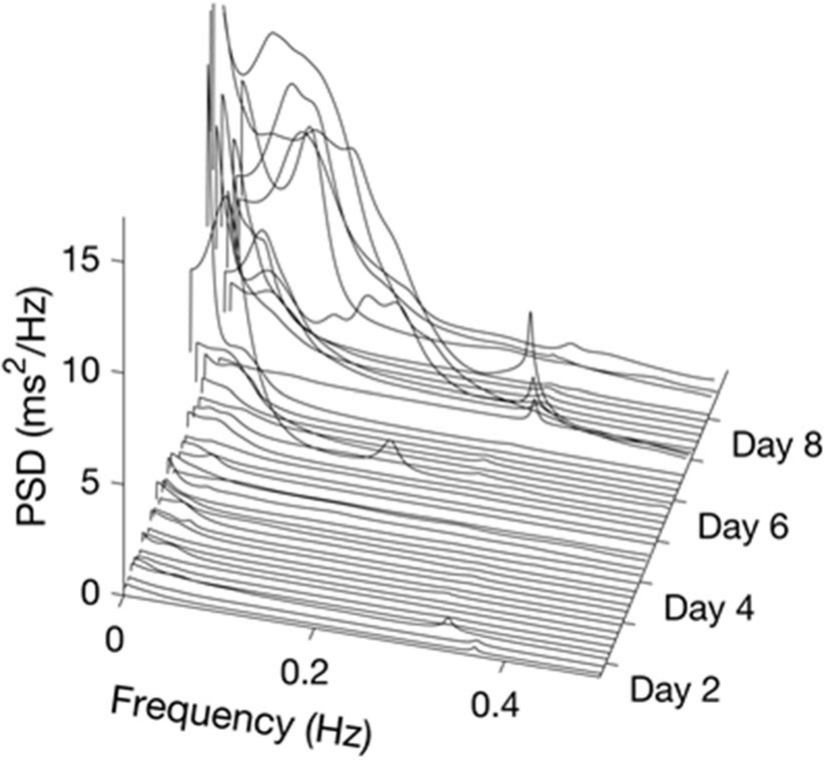
Power spectral density (PSD) of heart rate variability (HRV) for successive 4 h periods from day 2 to day 10 in the ICU. For each 4 h period, HRV spectra were first calculated for all 2-min periods where no or only few arrhythmic beats were present, and then the average PSD was determined

Figure 5 shows changes in heart rate, as represented by the mean RR interval, and the frequency of arrhythmic heart beats during day 2 to 10 in the ICU. Figure 6 shows the HRV power spectrum over the same time period, where the mean power spectrum was calculated for the same 4 h periods as for ICP, MAP and CPP. During some time periods the distance between density lines in the spectrum is larger than 4 h, indicating that there were not enough 2-minute intervals without arrhythmias to obtain a proper 4 h estimate of the spectral density. From the start of high frequency data recording (day 2 in the ICU) and until day 6 HRV was nearly absent. From approximately day 6 to day 8 there was a successive increase of the power in the very low-frequency region (VLF) near 0.05 Hz. A notable peak in the low-frequency region (LF, around 0.10 Hz) became present from day 8 until the end of recording on day 10. Finally, from day 2 to day 8 there was a small high frequency (HF) component around 0.3 Hz, which corresponded to the ventilator frequency. The HF peak increased in magnitude at day 8, corresponding to the timepoint where the patient’s consciousness increased, and spontaneous assisted ventilation was established.

**Fig. 7:**
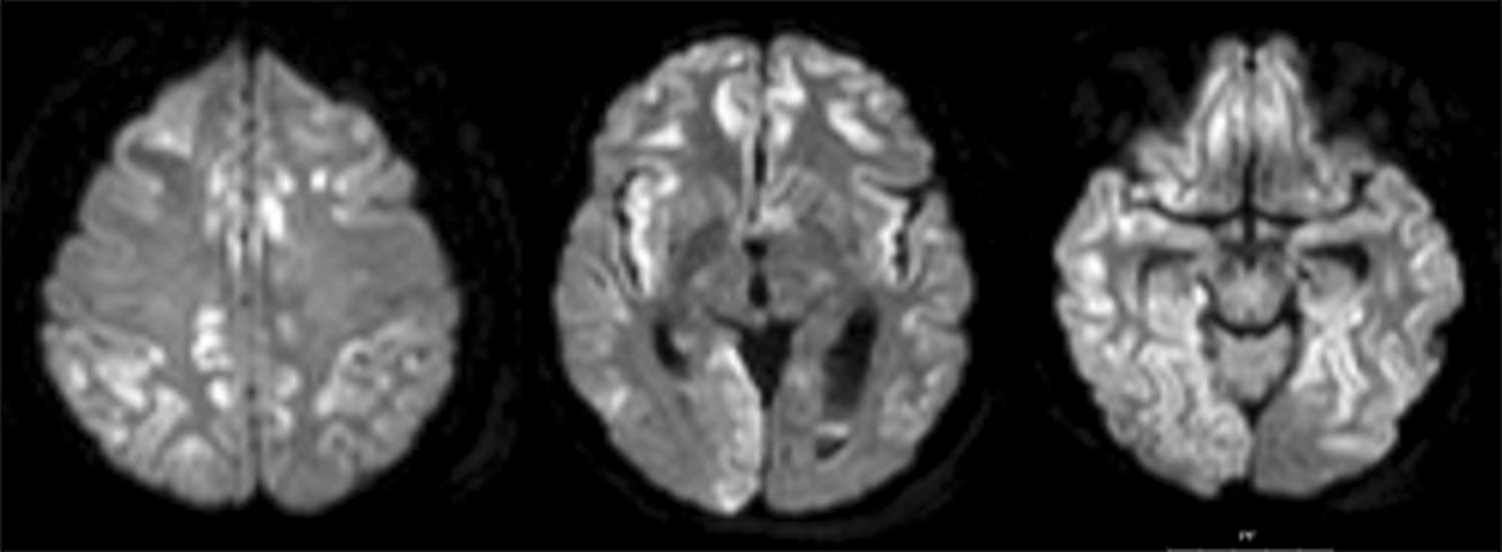
A diffusion weighted MRI on day 11 after admission showing scattered ischemic cortical lesions

A tracheostomy was performed on day 8, and all sedatives were stopped. On day 11 a diffusion weighted MR was performed which revealed scattered bilateral cortical ischemia (Fig. 7). Electroencephalography (EEG) showed post-anoxic-ischemic alpha-coma. Nutrition was kept to a minimum of caloric intake for 5 days (10 kcal/kg/day) according to the NICE guidelines, and then increased cautiously. After two weeks eyes opened spontaneously, finger movement was present in one hand, but the patient did not obey commands or give any contact. The patient returned to the primary hospital after 15 days, was eventually admitted to a rehabilitation center, and could thereafter return to home with assistance, being able to eat and talk.

The patient developed spastic paraparesis, epilepsy, and cognitive impairment. Over the 12-months after RFS the patient gradually developed a hydrocephalus and received a ventriculo-atrial shunt. ICP was measured during surgery and it was only 5 mmHg. No substantial recovery was seen after shunt surgery and the patient was admitted to hospital several times due to decreased consciousness without finding any new reasons for the condition. The patient died two years after the primary event.

## Discussion

In the present case report of a patient with severe RFS, course of disease, treatment and high-frequency multimodal monitoring data are presented. Similar data has, to our best knowledge, not been previously published. The report high-lights the severity of this condition, shows the inconsistency in cerebrovascular indices and is a reminder of how important the recognition of patients at risk for RFS, or patients with established RFS is [11]. It also empathizes the importance of proper initial treatment with phosphate delivery and low caloric nutrition.

This patient had three risk factors for RFS at primary admission: previous bariatric surgery with extensive weight loss, anorectic behavior and no caloric intake seven days before admittance. Even so parenteral nutrition was initially initiated without reduction of calories or substitution of phosphate. In two recent studies, calorie restriction improved survival RFS [12, 13], and calorie restriction has been recommended for patients at risk for RFS, or patients with established RFS [6]. The patient developed symptoms of RFS after 3 days, which is consistent with previous studies of symptom onset [14]. The condition described can be compared to an internal severe neurotrauma. Cerebral oedema in RFS is not previously described to our knowledge, even though encephalopathy is reported as a possible consequence [3, 5, 15, 16]. One may speculate that the brain edema and cerebral ischemia would have been less substantial if initial treatment had followed the NICE guidelines. Despite severe cerebral and cardiovascular deterioration, the patient survived after receiving maximal neuro-intensive care treatment. In such a severe condition, multimodal monitoring offers important guidance in the care of the patient.

This patient had a high to very high ICP initially, probably because of a generally developing brain edema. High ICP, around 20 mmHg, in itself seems to be a prognostic indicator for bad clinical outcome [17] and has been demonstrated to be associated with bad outcome in trauma patients [18, 19], patients with subarachnoid hemorrhage [20] and patients with bacterial meningitis [21, 22]. In our patient, the increased ICP in combination with cardiovascular instability and arterial hypotension during the first days resulted in periods with low CPP. Most probably this elicited persistent brain damage since ICP and CPP are the main parameters besides direct cerebrovascular injuries that affect the cerebral blood flow.

Cerebrovascular pressure reactivity is part of the cerebral autoregulation, and it reflects the capacity of cerebral arteries and arterioles to react to changes in transmural pressure. It may be assessed using the pressure reactivity index (PR_x_) [9], in which MAP is correlated against ICP to indicate an active or passive vasoconstriction in response to changes in MAP. However, some studies also indicate that PR_x_ may be affected by many other factors apart from pressure reactivity, such as alterations in temperature [23], arterial glucose concentration [24] and red blood cell transfusion [25]. Generally, negative PR_x_ values reflect preserved pressure reactivity while the more positive PR_x_ is, the more disturbed the autoregulation is considered to be. Critical values of PR_x_ have been described, where a threshold of 0.25 has been shown to maximize the prediction of survival, whereas a threshold of 0.05 predict favorable outcome with the highest probability [26]. One may speculate that in a seriously ill patient with cerebral edema, high ICP and low CPP, the cerebrovascular autoregulation is likely to be affected. This should be reflected in parameters applied to assess autoregulation, but according to PR_x_ (Fig. 3a) this patient had an intact cerebrovascular pressure reactivity from day 2 to 8 when treated in the ICU. On day 9 and start of day 10, PR_x_ increased considerably, indicating a disturbed cerebrovascular autoregulation. This was not in line with the patient’s clinical progression, and this finding is most likely related to the reduced sedation at this point. When looking at PR_x_ in more detail (Fig. 3b), a reduced pressure reactivity could be seen during a few 4-hour periods when the index was close to or higher than 0.2. A PR_x_ > 0.2 for a period of six hours or more has been found to be a strong indicator for mortality [27]. In our patient, even though PR_x_ corresponded to a compromised pressure reactivity a few times, most of the time, and as a mean over the whole monitoring period, PR_x_ indicated an intact autoregulation (-0.06 ± 0.18, mean ± SD). Considering the severe state of this patient these results are conflicting, and it puts the suitability of indirect measurements of cerebral autoregulation based on the PR_x_ index in question regarding RFS patients with cerebral engagement.

The RAP index has been introduced as a descriptor of the pressure-volume compensatory capacity, i.e. compliance, in patients with traumatic brain injury (TBI) [28, 29] and it is also applied for characterizing the cerebral compensatory reserve in patients with hydrocephalus [30]. A RAP index close to zero while still having a low ICP corresponds to a preserved intracranial compliance, since there is no correlation between a change in ICP pulse wave amplitude and mean ICP, while a RAP index approaching + 1 or negative values indicates a compromised compliance and an exhausted compensatory reserve respectively [29]. In our patient the RAP index varied between zero and 0.5 with a mean of 0.23. This could be compared to a study investigating the effect of decompressive craniectomy on ICP and cerebrospinal compensation following TBI, where the median (interquartile) RAP index of 27 patients with TBI was 0.4 (0.33;0.68) prior to, and 0.14 (0.12; 0.22) after decompressive craniectomy [31]. Additionally, in a study investigating 358 patients with moderate-to-severe TBI, the mean RAP was 0.638 ± 0.208 [32]. Thus, this severely ill RFS patient seemed to have a compensatory reserve varying within the interval from normal to low throughout the entire monitoring time, without signs of complete exhaustion, and the overall trend in RAP, from day 2 to day 10, was decreasing. Since ICP also decreased during the same time period this implies that the compensatory reserve improved slightly during the treatment period.

Multi-organ failure (including lung, liver and kidney disturbances) was displayed by this RFS patient. This is considerably different from patients with e.g. TBI, suffering from isolated brain injury or multi-trauma injury, where PR_x_ and RAP are most often applied. The patient was also very sensitive to manipulations, a sign which indicates a low cerebral compliance, and this enhances the questioning of the RAP index as an indicator of true compliance in this type of patient. We believe that the unexpected cerebrovascular manifestations presented here underlines the need for cautiousness when interpreting indexes of cerebrovascular autoregulation and compensatory capacity in the ICU, since the manifestation could be disease specific and dependent of type of injury.

Prolonged QT-time as well as parasympathetic/sympathetic imbalance affecting HRV have been described in patients with anorexia nervosa [33, 34]. However, results are conflicting describing both parasympathetic and sympathetic dominance [35]. Our patient was initially hemodynamically unstable, but was stabilized when the HRV recording started on day 2. The lack of HRV during day 2 to day 6 in the ICU could reflect a disturbed autonomic regulation. However, a loss of HRV has also been reported during general anesthesia [36], when the sympathetic nervous activity is markedly reduced. In our patient, there were nearly parallel changes in HRV and sedation level. From day 6 and on, the sedation level was gradually reduced, which was also when the activity in the VLF component started to increase. On approximately day 8, an LF component appeared and the HF component increased in magnitude. This was also the day when extra sedation was given due to increased alertness resulting in increased ICP and MAP. From day 9 and on, the sedation was stopped and HRV was also markedly increased. Since no HRV was recorded after day 10, we could not evaluate the parasympathetic/sympathetic balance when the patient was without sedation. Note that the changes in HRV were not due to cardiac arrhythmias, since there were a substantial number of 2-min periods without arrhythmias throughout the recording. Thus, we speculate that the changes seen in the HRV were related to the level of sedation as well as autonomous recovery.

## Conclusions

RFS is a rare and serious condition which may be difficult to recognize and may lead to death. Since multiple organ failure may occur, the initial treatment including correcting electrolyte disturbances and reduction of calorie intake is crucial. Serious deviations in homeostasis, high ICP levels, marked ECG abnormalities, as well as kidney and lung affections were observed. The generally normal manifestation of this patient’s cerebral autoregulation and compensatory reserve are difficult to explain due to the prevailing severe clinical state. It is therefore questionable if indirect measurements such as PR_x_ and RAP can be reliably applied during the neurointensive care treatment of RFS patients with cerebral engagement.
